# Revised Glycemic Index for Diagnosing and Monitoring of Diabetes Mellitus in South Indian Population

**DOI:** 10.7759/cureus.22510

**Published:** 2022-02-22

**Authors:** Rehana Khan, Janani Surya, Rupesh Agarwal, Tarun Sharma, Rajiv Raman

**Affiliations:** 1 Shri Bhagwan Mahavir Vitreoretinal Services, Sankara Nethralaya, Chennai, IND; 2 Ophthalmology, Tan Tock Seng Hospital, Singapore, SGP

**Keywords:** diabetes mellitus type 2, ethnicity, diabetic retinopathy, fasting plasma glucose, glycated haemoglobin (hba1c)

## Abstract

Aim: To find the optimal threshold of fasting plasma glucose (FPG) and glycated hemoglobin (HbA1c) for diagnosis of diabetes mellitus (DM) and to evaluate the association with diabetic retinopathy (DR) in the South Indian population.

Settings and Design: A retrospective population-based study.

Methods and Materials: A total of 909 newly detected type 2 DM patients were selected from our two previously conducted studies, which include an urban and a rural population of South India. All underwent estimation of fasting, postprandial plasma glucose (PPG), and other biochemical tests. A comprehensive and detailed ophthalmic examination was carried out. The fundi of patients were photographed using 45°, four-field stereoscopic photography. Based on receiver operating characteristic (ROC) curves, sensitivity and specificity were derived.

Results: The optimal cut-off values determined by maximizing the sensitivity and specificity of FPG and HbA1c using the Youden index were ≥ 6.17 mmol/L and ≥ 6.3%, respectively. By distributing the cut-off points into deciles and comparing them to the WHO criteria, we found that our HbA1c level of 6.60% was more than the WHO threshold (6.5%), with higher sensitivity (81.6%) and lower specificity (48.3%). The FPG level of 6.80 mmol/L was lower to the WHO criteria (7 mmol/L) with increased sensitivity (77.0%) and lower specificity (45.7%). Prevalence of DR by HbA1c levels between 6.5% and 6.9% was 15.3%. The prevalence of DR was more in the FPG category between 6.4 and 6.9 mmol/L and ≥ 7.5 mmol/L.

Conclusion: Our population-based data indicate that for the South Indian population HbA1c value of ≥63 % and FPG value of ≥6.17 mmol/L may be optimal for diagnosing DM with a high level of accuracy and will be useful for the identification of mild and moderate DR.

## Introduction

Type 2 diabetes mellitus (T2DM) is becoming more common all over the world [1]. It is most prevalent in developing countries, such as India, where 61.3 million people aged between 20 and 79 years were diagnosed with diabetes in 2011, and this number is expected to rise to 101.2 million by 2030 [2]. Fasting plasma glucose (FPG) or two-hour postprandial plasma glucose (PPG) test is the most widely used and traditional methods for diagnosing DM [3]. The cut-offs were determined based on the prevalence of microvascular complications in people with diabetes. The American Diabetes Association approved FPG 126 mg/dL (7.0 mmol/L) and glycated hemoglobin A1c (HbA1c) 6.5% in 2010 and was later recommended by the World Health Organization (WHO) for diagnosing DM [4]. This cut-off was determined based on findings from several studies [5-6] that showed a sharp increase in the prevalence of DM-related microvascular complications, such as diabetic retinopathy (DR) [7].

It is questionable whether the WHO recommended HbA1c of ≥ 6.5% and FPG of ≥7.0 mmol/L can effectively represent variations in the risk of DR in the Indian population due to ethnic variances. A population-based data reported by Mohan et al. suggested that HbA1c cut-off values of 6.1% and 6.4% are optimal for identifying DM in Asian Indians by PPG and FPG criteria, respectively [8]. However, this study had both newly diagnosed and people with known DM. There might be an influence of hypoglycemic medications on these cut-offs.

The WHO's cut-off values were predicated on the occurrence of microangiopathies. All of these conclusions were drawn from western literature. The question we wanted to answer was if these cut-offs still apply to the Indian population. As a result, the purpose of this study was to establish precise cut-off values for various glycemic markers, as well as to evaluate the sensitivity and specificity of current HbA1c and FPG criteria in a population-based sample of newly diagnosed DM patients.

## Materials and methods

**Figure 1:**
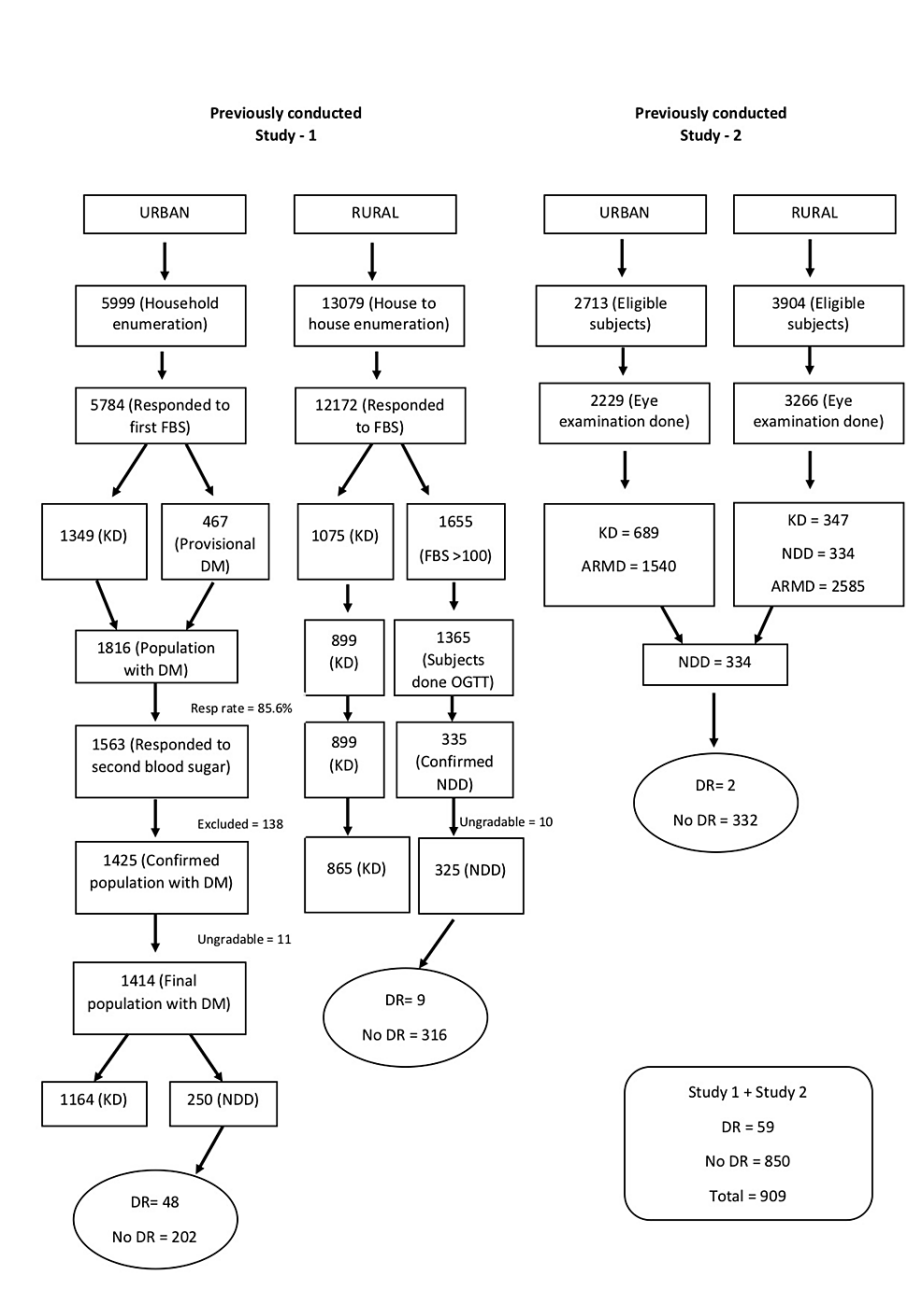
Flow chart showing a step-by-step enrolment and participation of subjects in this study population. FPG, fasting plasma glucose; HbA1c, glycated hemoglobin A1c; DM, diabetes mellitus; DR, diabetic retinopathy; KD, known diabetes; NDD, newly diagnosed diabetes; OGTT, oral glucose tolerance test; ARMD, age related macular degeneration

It is a retrospective population-based study, in which a total of 909 newly detected type 2 DM (T2DM) patients were selected from our two studies which followed the same protocol for assessment of DM and DR, done between 2007 and 2011, and included both urban and rural populations of South India. The study design and research methodology have been described in detail in our previous report [9]. To summarize, the urban population was studied in Chennai, while the rural population was studied in the rural portions of Tamil Nadu's Kanchipuram and Thiruvallur districts [10-11]. Using a multistage random cluster sampling procedure, a total of 5999 people from the urban population and 13079 people from the rural population, all over the age of 40, were identified. Step-by-step enrolment and participation of subjects in this study population are shown in Figure 1.

People with DM were identified based on the WHO criteria. All patients with known or provisional diabetes (first FBS ≥110 mg/dL) were referred to our hospital for a complete assessment that included a second FPG measurement and biochemical tests. Provisional DM patients were told to attend fasting on the day of the examination. All patients provided a full history prior to a complete eye examination, including demographics, DM history and comorbidities, and relevant ocular history. All the subjects underwent a detailed ophthalmic evaluation, anthropometric measurements, and biochemical tests. The details of these tests are mentioned elsewhere [11].

After pupillary dilation, retinal photographs were obtained using a Carl Zeiss fundus camera. All the patients underwent stereoscopic digital photography with a 45° field of view (posterior pole, nasal, superior, and inferior). Additional 30°, seven-field stereo digital pairs were collected for individuals who showed any signs of DR. The grading was done in a masked form by two independent ophthalmologists using the Klein et al. classification [12], and the grading agreement was high (k=0.82). The study was approved by the Institutional Review Board (Ethics committee) at Vision Research Foundation, Chennai and the informed consent was obtained from the subjects or if subjects were under 18, from a parent and/or legal guardian as per the Declaration of Helsinki. All the experiments were performed in accordance with relevant guidelines and regulations as approved by the Ethics committee.

Statistical analysis

The SPSS (version 21.0) (IBM Corp., Armonk, NY) was used for statistical analysis. Continuous variables with a normal distribution were described by their mean (standard deviation) value. Numbers (percent) were used to show categorical data. Comparisons between the mean values were performed using Student t-tests, whereas the median values were performed using the Mann-Whitney test. For the analyses, FPG and HbA1c were categorized by deciles. For each glycemic metric, receiver operating characteristic (ROC) curves were developed to compute and compare the area under the curves and to discover the best cut-offs for detecting DR by utilizing the Youden index to maximize sensitivity and specificity. We assessed the sensitivity, specificity, positive predictive value (PPV), negative predictive value (NPV), and accuracy of various cut-off values by cross-tabulating the prevalence of DR against the HbA1c and FPG categories. Differences between two independent means were calculated (two-sided test; p<0.05) and a post hoc test was done to calculate power analysis.

## Results

A total of 19,078 people [rural (n=13079) and urban (n=5999)] of previously conducted DR study 1 and a total of 6617 [rural (n=3904) and urban (n=2713)] previously conducted DR study 2 were identified. 

In DR study 1, 1414 people with diabetes were identified from a total of 5999 people in the urban population, with 1164 were known DM and 250 were newly diagnosed DM. From 250 newly diagnosed DM, 48 were confirmed with DR and no DR changes were noted in 202 individuals. In a rural population of 13079 individuals, 1190 participants (865, known DM; 325, newly diagnosed DM) were analyzed. Of 325 newly diagnosed DM, nine were confirmed with DR, and 316 does not show any DR changes. In an urban population of DR study 2, 689 were known DM and in rural population, 347 were known DM and 334 were newly diagnosed DM. In 334 newly diagnosed DM, two were diagnosed with DR, and 331 showed no changes of DR. 

Thus a total of 909 newly detected T2DM were analyzed for this study. Some 59 individuals showed DR changes and 850 individuals showed no changes in DR (Figure 1). 

The characteristics of patients with and without retinopathy are compared in Table 1. Patients with DR were younger, women were more impacted, and the DR group had high levels of total cholesterol, low density, and high-density lipoproteins than those without DR, however, the differences were not statistically significant. The DR group's FPG and HbA1c were both considerably greater than the non-DR group's. Other factors such as systolic and diastolic blood pressure, as well as body mass index, were similar amongst the groups. Smoking, non-alcohol use, and a lesser level of education were all associated with being in the DR group.

**Table 1:** Characteristics of the participants divided according to the presence of DR. Continuous data are presented as means (means ± standard deviation, SD), and categorical data are presented as proportions n(%). DR, diabetic retinopathy; BMI, body mass index; SBP, systolic blood pressure; DBP, diastolic blood pressure; FPG, fasting plasma glucose; HbA1c, glycated hemoglobin A1c; TC, total cholesterol; HDL, high density lipoprotein; LDL, low density lipoprotein

Characteristics (n=909)	No DR (n=850)	DR (n=59)	p
Age (years)	57.4 ± 10.2	55.42 ± 10.58	0.15
Sex			
Male (%)	380 (44.71)	21 (35.59)	0.17
Female (%)	470 (55.29)	38 (64.41)	
BMI (kg/m2)	24.00 ± 4.77	23.67 ± 4.24	0.6
SBP (mmHg)	128.27 ± 18.66	124.14 ± 16.50	0.09
DBP (mmHg)	81.95 ± 11.31	79.42 ± 9.24	0.09
FBG (mmol/L)	8.11 ± 2.91	13.17 ± 4.37	<0.0001
HbA1c (%)	7.08 ± 1.71	10.42 ± 1.41	<0.0001
TC (mg/dl)	179.48 ± 40.31	186.63 ± 37.02	0.18
HDL (mg/dl)	40.94 ± 11.14	43.07 ± 11.50	0.15
LDL (mg/dl)	110.04 ± 33.36	112.67 ± 34.22	0.55
Education			
Illiterate	398 (46.82)	21 (35.59)	0.23
Primary education	165 (19.41)	13 (22.02)	
Secondary and above	287 (33.76)	25 (42.37)	
Smoking status			
Yes	601 (70.71)	49 (83.05)	0.04
No	249 (29.29)	10 (16.95)	
Alcohol consumption			
Yes	391 (46.00)	15 (25.42)	0.002
No	459 (54.00)	44 (74.58)	

**Figure 2:**
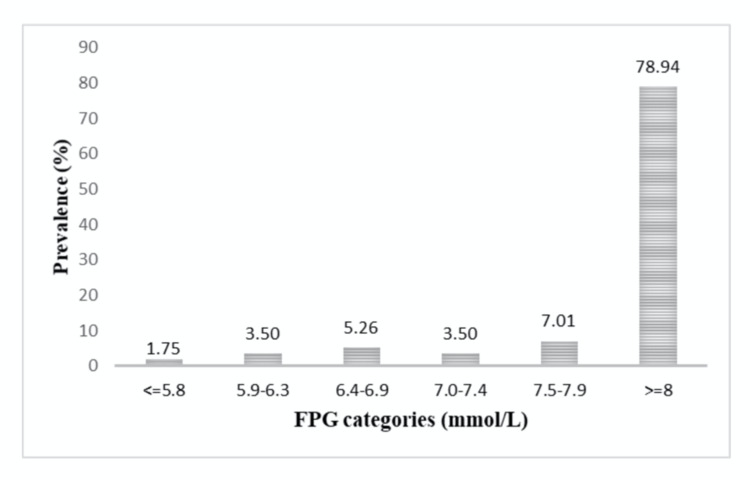
Prevalence of DR by FPG categories. DR, diabetic retinopathy; FPG, fasting plasma glucose

**Figure 3:**
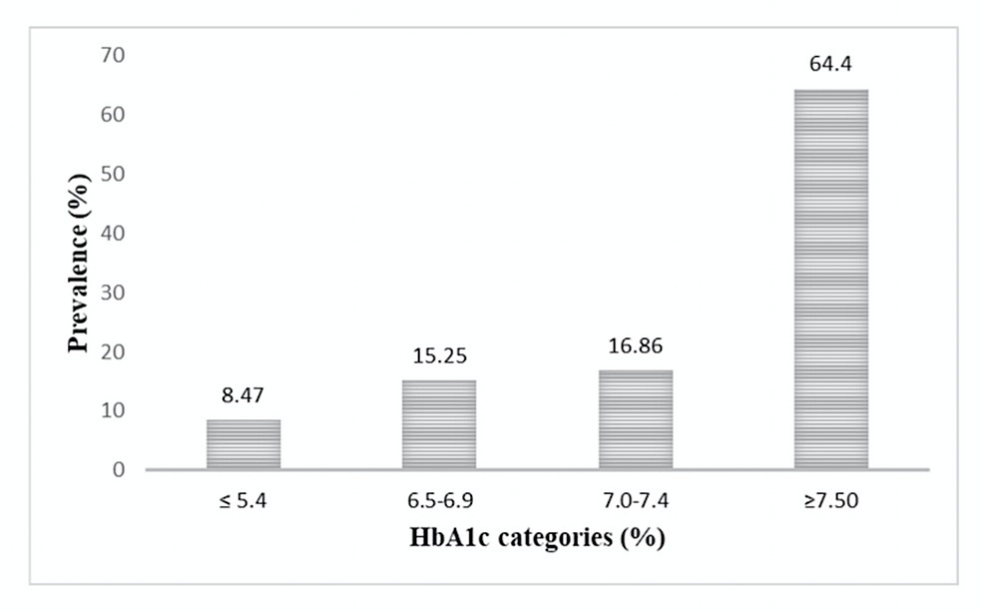
Prevalence of DR by HbA1C categories. DR, diabetic retinopathy; HbA1C, glycated hemoglobin A1C

**Figure 4:**
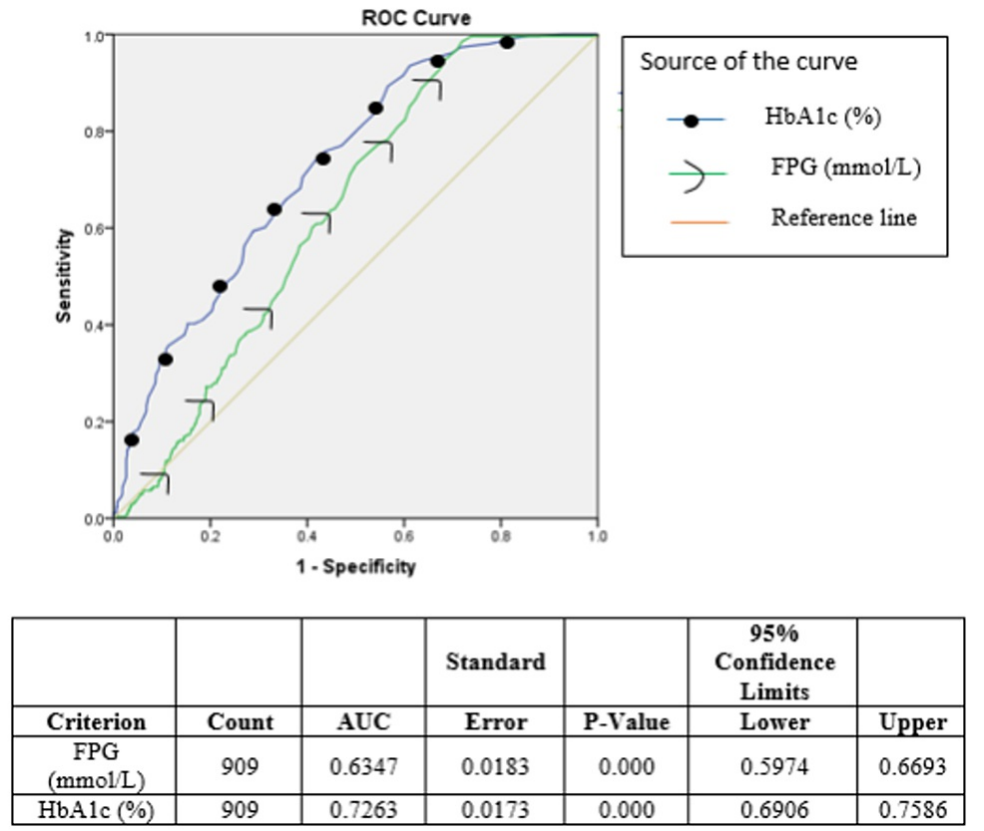
ROC curves of HbA1c and FPG for detecting DR in 909 newly detected diabetes subjects. ROC, receiver operating characteristic; HbA1C, glycated hemoglobin A1c; FPG, fasting plasma glucose; DR, diabetic retinopathy

Figures 2-3 show the prevalence of DR by HbA1c and FPG categories. The prevalence of DR increased with increasing categories of HbA1c. Prevalence between HbA1c levels of 6.5% and 6.9% was 15.3%. The prevalence of DR is more in the FPG category between 6.4 and 6.9 mmol/L and ≥ 7.5 mmol/L. The area under the ROC curves for FPG and HbA1c were 63.5% [95% confidence interval (CI) 59.7, 66.9] and 72.6% [95% confidence interval (CI) 69.1, 75.9] respectively, with a significant difference in the potential to predict DR (p<0.001) (Figure 4).

The sensitivity, specificity, PPV, NPV, and accuracy at different FPG and HbA1c cut-off values are shown in Tables 2-3. In the overall 909 participants, the ideal cut-off values were 6.17 mmol/L and 6.3 percent, respectively, as calculated by maximizing the sensitivity and specificity of FPG and HbA1c using the Youden index.

**Table 2:** Cut-off values of FPG defined by sensitivity and specificity. FPG, fasting plasma glucose; PPV, positive predictive value; NPV, negative predictive value

Cut-off value (mmol/L)	Sensitivity (%)	Specificity (%)	PPV (%)	NPV (%)	Accuracy (%)	Youden Index
5.83	100%	17%	33%	99%	41%	0.1659
5.89	100%	19%	33%	99%	42%	0.1829
5.94	100%	21%	34%	99%	43%	0.2045
6	100%	22%	34%	99%	45%	0.2199
6.06	100%	24%	35%	99%	46%	0.24
6.11	100%	26%	35%	99%	47%	0.257
6.17	98%	28%	36%	98%	48%	0.2655
6.22	97%	29%	35%	96%	49%	0.2595
6.28	95%	32%	36%	94%	50%	0.2627
6.33	93%	33%	36%	92%	50%	0.2544
6.39	90%	35%	36%	90%	51%	0.253
6.44	89%	36%	36%	89%	51%	0.2531
6.5	87%	38%	36%	87%	52%	0.2424
6.56	85%	39%	36%	87%	52%	0.2395
6.61	82%	40%	36%	85%	52%	0.2219
6.67	81%	41%	36%	84%	53%	0.222
6.72	78%	44%	36%	83%	53%	0.2168
6.78	78%	45%	36%	83%	54%	0.2238
6.83	77%	46%	36%	83%	55%	0.2269
6.89	75%	47%	37%	83%	55%	0.2286
6.94	74%	49%	37%	82%	56%	0.2302
7	74%	50%	37%	82%	56%	0.231
7.06	73%	50%	37%	82%	57%	0.2295
7.11	71%	51%	37%	81%	57%	0.2212
7.17	68%	52%	36%	80%	57%	0.2036
7.22	66%	53%	36%	80%	57%	0.1906
7.28	64%	54%	36%	79%	57%	0.1831
7.33	64%	55%	36%	79%	58%	0.1885
7.39	62%	56%	36%	78%	58%	0.1801
7.44	61%	57%	36%	78%	58%	0.1802
7.5	61%	58%	37%	79%	59%	0.1894

**Table 3:** Cut-off values of HbA1c defined by sensitivity and specificity. HbA1c, glycated hemoglobinA1c; PPV, positive predictive value; NPV, negative predictive value

Cut-off value (%)	Sensitivity (%)	Specificity (%)	PPV (%)	NPV (%)	Accuracy (%)	Youden Index
5	100%	7%	30%	100%	34%	0.0725
5.1	100%	12%	31%	99%	37%	0.1196
5.2	100%	14%	32%	99%	39%	0.1381
5.3	99%	17%	33%	98%	41%	0.1636
5.4	99%	19%	33%	98%	42%	0.1768
5.5	98%	20%	33%	97%	43%	0.1868
5.6	98%	22%	34%	97%	44%	0.2031
5.7	97%	28%	35%	96%	48%	0.2571
5.8	96%	30%	36%	95%	49%	0.2626
5.9	95%	33%	36%	95%	51%	0.2827
6	95%	36%	37%	94%	53%	0.3044
6.1	93%	39%	38%	94%	55%	0.3238
6.2	92%	40%	38%	92%	55%	0.3169
6.3	89%	43%	39%	91%	57%	0.3264
6.4	87%	44%	39%	89%	57%	0.3126
6.5	84%	46%	38%	88%	57%	0.2974
6.6	82%	48%	39%	87%	58%	0.2991
6.7	79%	51%	39%	86%	59%	0.2993
6.8	77%	53%	40%	85%	60%	0.2994
6.9	76%	56%	41%	85%	62%	0.3219
7	74%	58%	42%	85%	63%	0.3228
7.1	71%	61%	42%	84%	64%	0.3145
7.2	68%	61%	42%	83%	63%	0.2962
7.3	66%	64%	43%	82%	65%	0.3025
7.4	64%	66%	43%	82%	65%	0.2988
7.5	60%	69%	44%	81%	66%	0.2883

Based on the decile distribution and ROC curves of our current study, the sensitivity and specificity of several cut-off values for diagnosing DR were compared to the existing WHO diagnostic criteria (Table 4).

**Table 4:** Glycemic cut-off points derived from different analytic methods and the WHO criteria. FPG, fasting plasma glucose; HbA1c, glycated hemoglobin A1c; ROC, receiver operating characteristics; WHO, World Health Organization; 2hPG, 2-hour post load plasma glucose

	Cut-off values	Sensitivity	Specificity
Decile distribution			
FPG (mmol/L)	6.8	0.77	0.457
HbA1c (%)	6.6	0.816	0.483
ROC curve analysis			
FPG (mmol/L)	6.17	0.985	0.281
HbA1c (%)	6.3	0.893	0.434
WHO criteria			
FPG (mmol/L)	7	0.65	0.905
2hPG	11.1	0.353	0.917
HbA1c (%)	6.5	0.625	0.995

In the current study, the HbA1c level of 6.60 for diagnosing DM was marginally higher than the WHO criteria of 6.5% [7], with relatively increased sensitivity (81.6%) and lower specificity (48.3%). The FPG level of 6.80 mmol/L for diagnosing DM was slightly lesser than the WHO criteria of 7 mmol/L [7], but it had higher sensitivity (77%) and poorer specificity (45.7%).

The prevalence of DM was compared using new and old cut-off values. There is a 16% rise in DM patients identified with the new cut-off value of FPG 6.17 mmol/L, and a 2.3% increase in DM patients identified with HbA1c 6.3%.

## Discussion

Type 2 DM is becoming a major public health problem all over the world, especially in developing countries like India, due to extremely rapid economic growth and changes in lifestyle and dietary factors [13]. Although the prevalence of DM has increased in recent years, diagnosis is often delayed until the appearance of microvascular complications. The diagnosis criteria for DM are currently based on lab values, with the cut-off value depending on microvascular issues, particularly DR [14]. A recent clinical guideline by the American College of Physicians (ACP) recommends the HbA1c level to be between 7% and 8% for treating patients with T2DM [15]. Several studies in different ethnic groups have attempted to re-evaluate diagnostic criteria and establish the most reliable cut-off values. Few studies suggested that the observed differences in HbA1c cut-points between ethnic groups are more likely due to biological differences in red blood cell life span or genetic variation, yet, only glycemic variants were associated with increased T2DM risk over a decade-long follow-up period [16]. In studies carried out between different ethnic groups, the HbA1c thresholds for raising the prevalence of any DR were reported to be between 6.1% and 7.0% [17-19, 5-6]. Few studies have determined the optimal cut-off points for HbA1c with maximum sensitivity and specificity. In Pima Indians [6], they found the HbA1c cut-off to be 7.0% for detecting any DR, and for the Japanese population, the optimal cut-off was found to be 5.7% [20]. The ideal HbA1c criteria for detecting mild and moderate DR, according to a South Asian study [20], were 49 mmol/mol (6.6%) and 53 mmol/mol (7.0%). The HbA1c cut-off for diagnosing DM was determined to be 42 mmol/mol (6.0%) in a French cohort study [21]. For the Southern Chinese population, the HbA1c cut-off for diagnosing DM was found to be 6.3% [22]. A recent study in the United States revealed that blacks are more likely than whites to develop DR at lower HbA1c levels, and advised that the HbA1c threshold for diagnosing DM in blacks be lowered [23].

In any case, the results are still conflicting. Several studies have found that the prevalence of DR has a glycemic limit. There has been a continuous discussion about whether to utilize ethnicity-explicit HbA1c cut-off values, which should be used to diagnose DM. The variation in optimal cut-off values could be because of test contrasts in estimating HbA1c [24-25]. The different ethnicities, age, sex distribution, and prevalence of DM could be other reasons for the variability in optimal cut-off values [19]. Based on DR sensitivity and specificity findings, diagnostic cut-offs for HbA1c in DM have been changed. The ADA recommended that the FPG cut-off point be reduced to 7.0 mmol/L from 7.8 mmol/L in 1997 since few studies had shown a linear increase in the prevalence of DR above this level. Based on the pooling investigation report, ADA proposed the HbA1c cut point of 6.5% for diagnosing DM and discovered the relationship of moderate and severe DR [26]. In patients with early-stage diabetes, relying entirely on the proposed HbA1c criterion for diabetes diagnosis could result in missed diagnoses and therapeutic interventions.

In this sample of the South Indian population, the relationship of HbA1c with microvascular complications including mild DR and moderate DR was found. Between the fourth and fifth deciles, the FPG and HbA1c thresholds for detecting DR were observed (FPG > 6.80 mmol/L and HbA1c > 6.6%). The ideal cut-offs for FPG and HbA1c were 6.17 mmol/L and 6.3%, respectively, according to ROC curve analysis. In this cohort, we observed that the present diagnostic criteria had a low specificity and a high sensitivity.

In our study, the HbA1c cut-off value of 6.3% and the FPG cut-off value of 6.17 mmol/L were found to be helpful in diagnosing DM. These values can help to differentiate between mild and moderate DR, as well as prevent retinal disorders caused by high blood glucose levels. Adherence to the suggested guidelines for the mild stage of DR is important since it will increase the prevalence of DR and ensure that patients do not progress to more serious stages.

Though the number of DR (n=59) subjects was less to derive cut-off values, the post hoc power calculation showed a power of 95%, which suggested that the study has sufficient power. The strength of this study is that it is population-based associated with an Asian Indian population, an ethnic group that has a high susceptibility to T2DM. A 45°, stereoscopic digital photography was used to capture the dilated fundus of the patients. The use of an ultrawide-field fundus camera, which can image 80% of the retinal surface would have given less possibility of missing any peripheral lesions of DR, which was not technically available until recent times. But still, an additional 30° were taken for participants with DR which is significant when predominantly peripheral lesions are associated [27]. The diagnosis of DR was made using Klein's method, and retinal photography was precisely interpreted by a qualified eye care physician. A standardized protocol was used to estimate the grading of DR and the addition of other microvascular endpoints. In addition, HbA1c measurements have been certified by the National Glycohemoglobin Standardization Program. As in our study, we had included only the newly diagnosed DM, the effect of hypoglycemic agents which could affect the cut-offs were avoided. 

## Conclusions

In conclusion, an HbA1c of ≥6.3% and an FPG of ≥6.17 mmol/L may be appropriate for diagnosing diabetes in the South Indian population. Microvascular problems have been linked to higher glycemic index values. The adoption of these cut-off values for the best diagnosis of those with mild and moderate retinopathy is supported by our findings. These cut-off points could be crucial in preventing retinal disorders caused by high blood sugar levels. Adherence to the prescribed guidelines for the mild stage of DR is critical since it will increase the prevalence of DR and ensure that patients do not progress to the more serious stages. Treatment of hyperglycemia and diabetic complications is much more expensive than preventing diabetes.
